# Case Report: Neurodevelopmental Outcome in a Small-for-Gestational-Age Infant With Symptomatic Hyperinsulinemic Hypoglycemia, Gaze Preference, and Infantile Spasms

**DOI:** 10.3389/fendo.2022.818252

**Published:** 2022-06-03

**Authors:** Suresh Chandran, Kok Wooi Teoh, Krishnappa Janardhan, Fabian Yap

**Affiliations:** ^1^ Department of Neonatology, KK Women’s and Children’s Hospital, Singapore, Singapore; ^2^ Pediatric Academic Clinical Programme, Lee Kong Chian School of Medicine, Singapore, Singapore; ^3^ Pediatric Academic Clinical Programme, Duke-NUS Medical School, Singapore, Singapore; ^4^ Pediatric Academic Clinical Programme, Yong Loo Lin School of Medicine, Singapore, Singapore; ^5^ Department of Pediatric Neurology , KK Women’s and Children’s Hospital, Singapore, Singapore; ^6^ Department of Pediatric Endocrinology , KK Women’s and Children’s Hospital, Singapore, Singapore

**Keywords:** infantile spasms, hyperinsulinemic hypoglycemia, gaze preference, parieto-occipital neuronal injury, diazoxide, hormone therapy, vigabatrin

## Abstract

Recurrent and profound hypoglycemia is a leading cause of neonatal brain injury. Small-for-gestational-age infants are at risk of hypoglycemia due to substrate deficiency and hyperinsulinism. Inappropriate insulin secretion by the β-cells of the pancreas results in hypoglycemia, neuronal energy deprivation, and parieto-occipital brain injury. Hypoglycemic neuronal injury is increasingly being identified as a trigger for infantile spasms, even though the underlying pathophysiological mechanisms remain elusive. A term, small-for-gestational-age male infant developed severe symptomatic hypoglycemia on day 3 of life. He required a high glucose infusion rate (14 mg/kg/min) to maintain normoglycemia. Critical blood samples showed inappropriate insulin levels while hypoglycemic and hypoketonemic, consistent with a diagnosis of hyperinsulinemic hypoglycemia. Blood glucose levels normalized with a diazoxide dose of 5 mg/kg/day. Gradually, glucose infusion was weaned with increasing oral feeds while maintaining prefeed capillary blood glucose levels. While at home, his glucose profile remained stable on the self-weaning dose of diazoxide. He passed a resolution fasting study at 4 months of age after weaning off diazoxide. He developed left gaze preference at 2.5 months of age while on treatment for hyperinsulinemic hypoglycemia but developed infantile spasms at 5 months that was confirmed with an electroencephalogram (EEG). Gaze preference may be epileptic, even in the absence of seizures. Spasms were well controlled with high-dose prednisolone therapy. At the age of 6 years, he has a mild fine motor delay and learning disabilities. Early diagnosis and treatment of infantile spasms have a better prognosis. Identifying gaze preference as a predating sign of occipital lobe epilepsy, EEG monitoring, and, if required, treatment could have possibly averted the genesis of infantile spasms.

## Introduction

Neonatal hypoglycemia is a common metabolic disorder, especially in infants at risk of hypoglycemia. They include infants of diabetic mothers, small for gestational age (SGA) and large for gestational age infants, and preterm infants (1). A failure of metabolic transition from placental to enteral nutrition leads to glucose dysregulation in such high-risk infants. Even though many hypoglycemic infants are asymptomatic, the residual brain injury can be significant (2). There is no consensus on the level of glucose or duration of hypoglycemia that could cause brain injury (3). SGA infants are particularly at risk due to hyperinsulinism (1). In hyperinsulinemic infants, the inappropriate persistence of insulin inhibits glycogenolysis, gluconeogenesis, and ketogenesis, depriving the brain of both primary (glucose) and secondary (ketones) energy sources, consequently causing neuroglycopenia (4). In symptomatic neonatal hypoglycemic infants, Burns et al. have reported a spectrum of severe forms of brain injuries, including cortical abnormalities (51%), white matter hemorrhages (30%), and basal ganglia lesions (40%) (5).

Infantile spasms (IS), an age-specific epilepsy syndrome manifesting in the first year of life, have many underlying causes, including structural, genetic, and idiopathic causes (6, 7). IS occur in clusters and are typically a sudden, brief, bilateral, and symmetric contraction of the neck, trunk, and limb muscles, with an onset peaking between 4 and 7 months of age. Neonatal hypoglycemic brain injury (NHBI) is a recognized trigger of IS and is emerging as an important acquired structural cause for IS after hypoxic–ischemic encephalopathy (3, 7). Approximately 60%–70% of IS due to NHBI respond to medical therapy (7). Gaze preference was reported as a preceding event or as a sole presenting sign of occipital lobe epilepsy and may be of significance in infants with established parieto-occipital injury. We discuss a term SGA infant who had severe symptomatic hypoglycemia on day 3 of life and had MRI evidence of NHBI. He had left lateral gaze preference at 2.5 months and presented with IS at 5 months of age. At 6 years, he has a mild neurodevelopmental delay and learning disabilities.

## Case Presentation

A term male asymmetric SGA infant with a birth weight of 2,295 g (38 weeks, 2nd percentile, −2.01 SDS) and head circumference of 33 cm (26th percentile, −0.64 SDS) was born to a primigravida mother *via* normal vaginal delivery. Parents were non-consanguineous and of Chinese origin. The mother had regular antenatal care, and her serologies were normal. Antenatal ultrasound scans showed fetal growth restriction in the 3rd trimester. Apgar scores were 9 at 1 and 5 min of life. The infant was discharged home on day 2 of life after establishing breastfeeding. On day 3, he was lethargic during a neonatal jaundice review at a primary care clinic. He was immediately referred to our tertiary care hospital for further assessment. However, he developed generalized seizures before he arrived at the emergency department.

**Figure 1:**
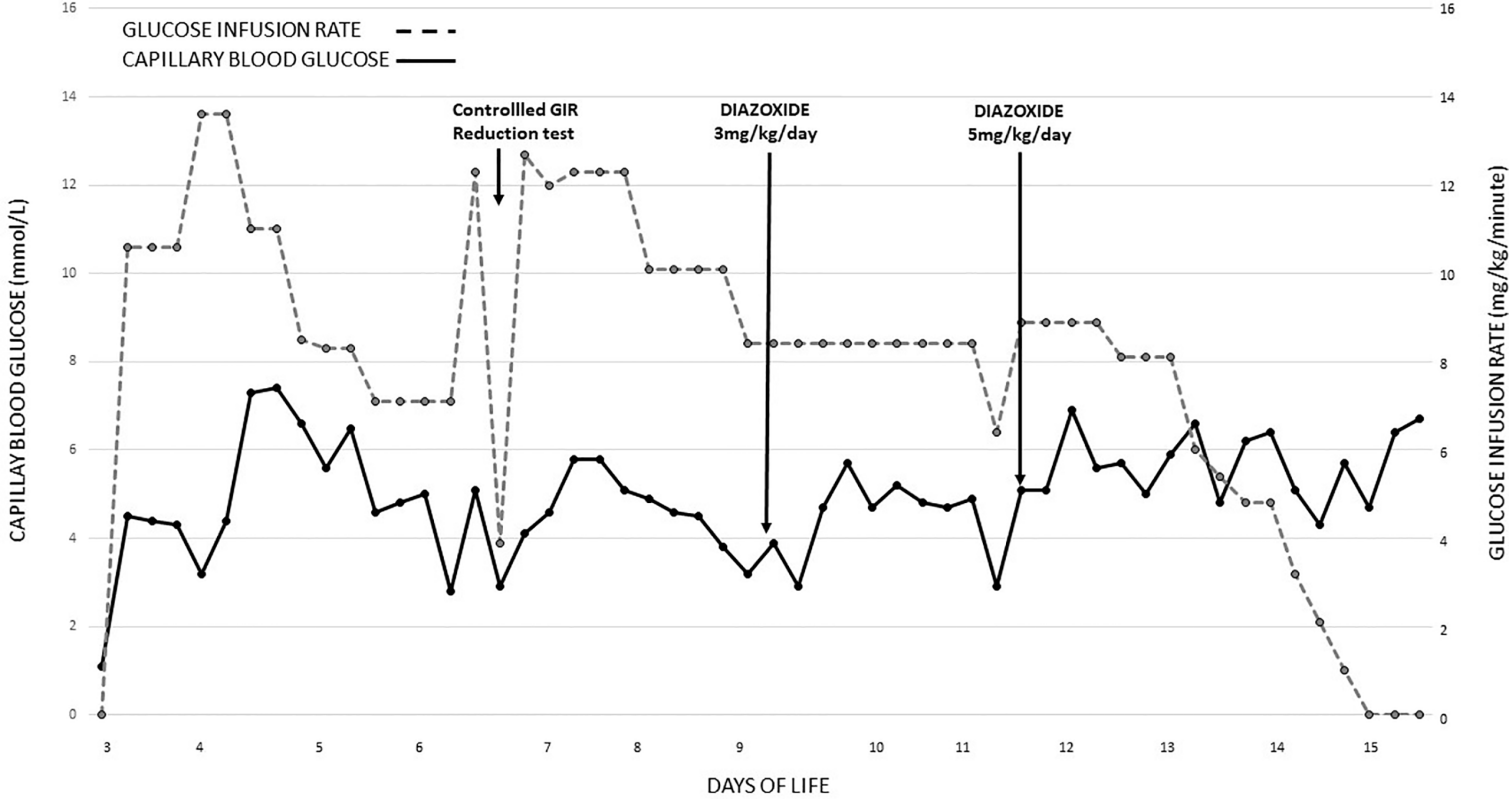
Blood glucose recordings and glucose infusion rates were required from day 3 of life till glucose control was achieved with diazoxide. GIR, glucose infusion rate.

On arrival, plasma glucose was 0.3 mmol/L (5.4 mg/dl), and his seizures were refractory to two slow dextrose boluses and a glucose infusion rate (GIR) of 8.4 mg/kg/min. He was intubated for airway protection. He eventually required a GIR of 14 mg/kg/min to achieve normoglycemia (3.5–7 mmol/L (63–126 mg/dl)). Investigations included arterial blood gas, full blood count, liver and renal function tests, and C-reactive protein, and all were unremarkable. Antibiotics were initiated. The blood culture was sterile after 48 h, and antibiotics were discontinued. A diagnosis of hyperinsulinemic hypoglycemia (HH) was considered given the high GIR in an SGA infant and was confirmed by a controlled GIR reduction test. HH diagnosis was made with an inappropriate insulin level of 8.6 mU/L with hypoketonemia (0.2 mmol/L) during a hypoglycemic episode (plasma glucose, 2.7 mmol/L (48 mg/dl)) with adequate growth hormone (64.2 µg/L) and cortisol (437 nmol/L) response. Inborn errors of metabolism screening by tandem MS, serum ammonia (47 μmol/L), and lactate (2.2 mmol/L) were within normal limits. He was weaned from ventilatory support on day 8 of life. Diazoxide was initiated on day 9 of life at a dose of 3 mg/kg/day in 2 divided doses with diuretics, and the diazoxide was increased to 5 mg/kg/day to achieve optimal glucose levels (**Figure 1**). Intravenous GIR was weaned, while a stepwise increment of milk feeds was allowed. Pre-feed capillary blood glucose remained stable on full feeds, and he passed a 6-h safety fasting study before home on day 17 of life. Being diazoxide-responsive HH, genetic testing was not done. He remained on home glucose monitoring under the guidance of our hypoglycemia team. Diazoxide was gradually weaned off and eventually discontinued at 4 months of age. An appropriate β-cell response to glucose was demonstrated by an 8-h resolution fasting study. Continued glucose testing during an intercurrent illness was advised.

**Figure 2:**
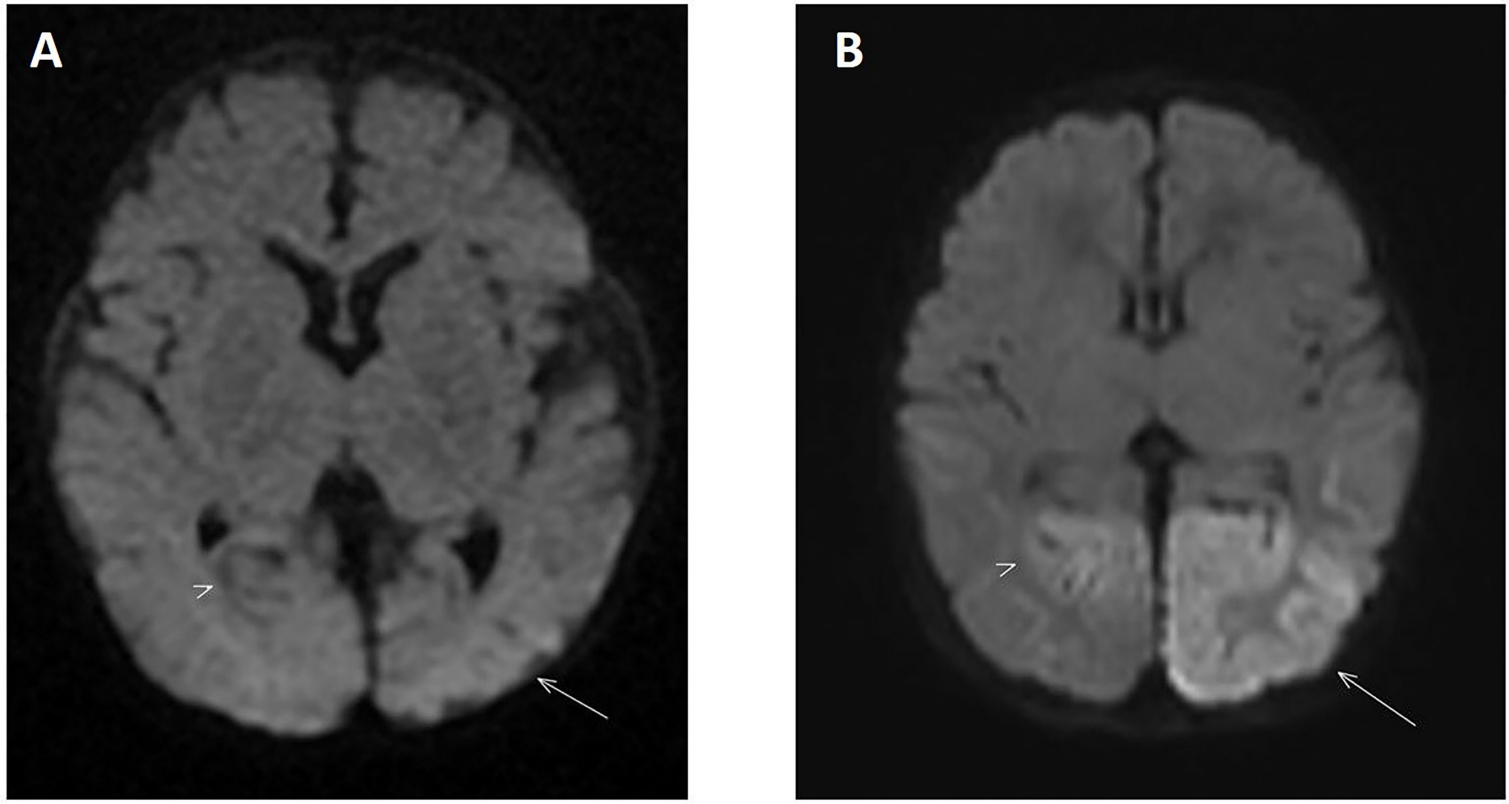
**(A)** Diffusion-weighted MRI on day 4 of life showing increased signal intensity located in the left occipital lobe (arrow) consistent with an area of restricted diffusion. The right occipital lobe is also involved to a lesser extent (arrowhead). These findings are consistent with severe symptomatic hypoglycemia when correlated to clinical and laboratory investigations. **(B)** Diffusion-weighted MRI performed 2 months later shows resolution of the restricted diffusion in the previously involved areas. However, there is now residual atrophy in both occipital lobes with the left side more severely involved compared to the right (arrow and arrowhead respectively).

MRI of the brain on day 4 of life demonstrated areas of high T2 and T2 fluid-attenuated inversion recovery (FLAIR) signal intensity with restricted diffusion in the splenium of the corpus callosum, the gyri of the left occipital lobe, and, to a lesser extent, the left parietal lobe and cuneus of the right occipital lobe. These findings are compatible with cytotoxic cerebral edema, consistent with neonatal hypoglycemic encephalopathy (**Figure 2A**). At 2 months of age, a repeat MRI showed several foci of T1 hyperintensity in the gyri of the left occipital and parietal lobes, representing cortical laminar necrosis. Gliosis was also noted in both occipital lobes, with the left side more severely affected than the right (**Figure 2B**). Parents were counseled regarding the long-term neurodevelopmental outcome and the need for close follow-up in the background of neuronal injury.

**Figure 3:**
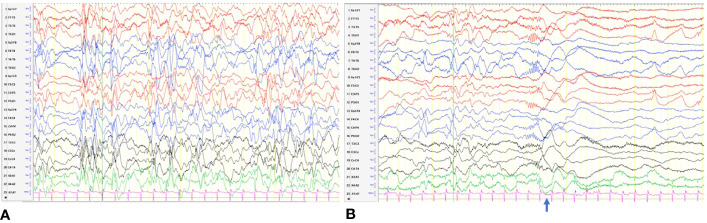
**(A)** Hypsarrhythmia: discontinuous, asynchronous background with high-voltage irregular slow waves and multifocal spikes. **(B)** An electrodecremental event. The clinical spasm (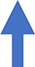) was associated with sudden attenuation in the voltage.

**Figure 4:**
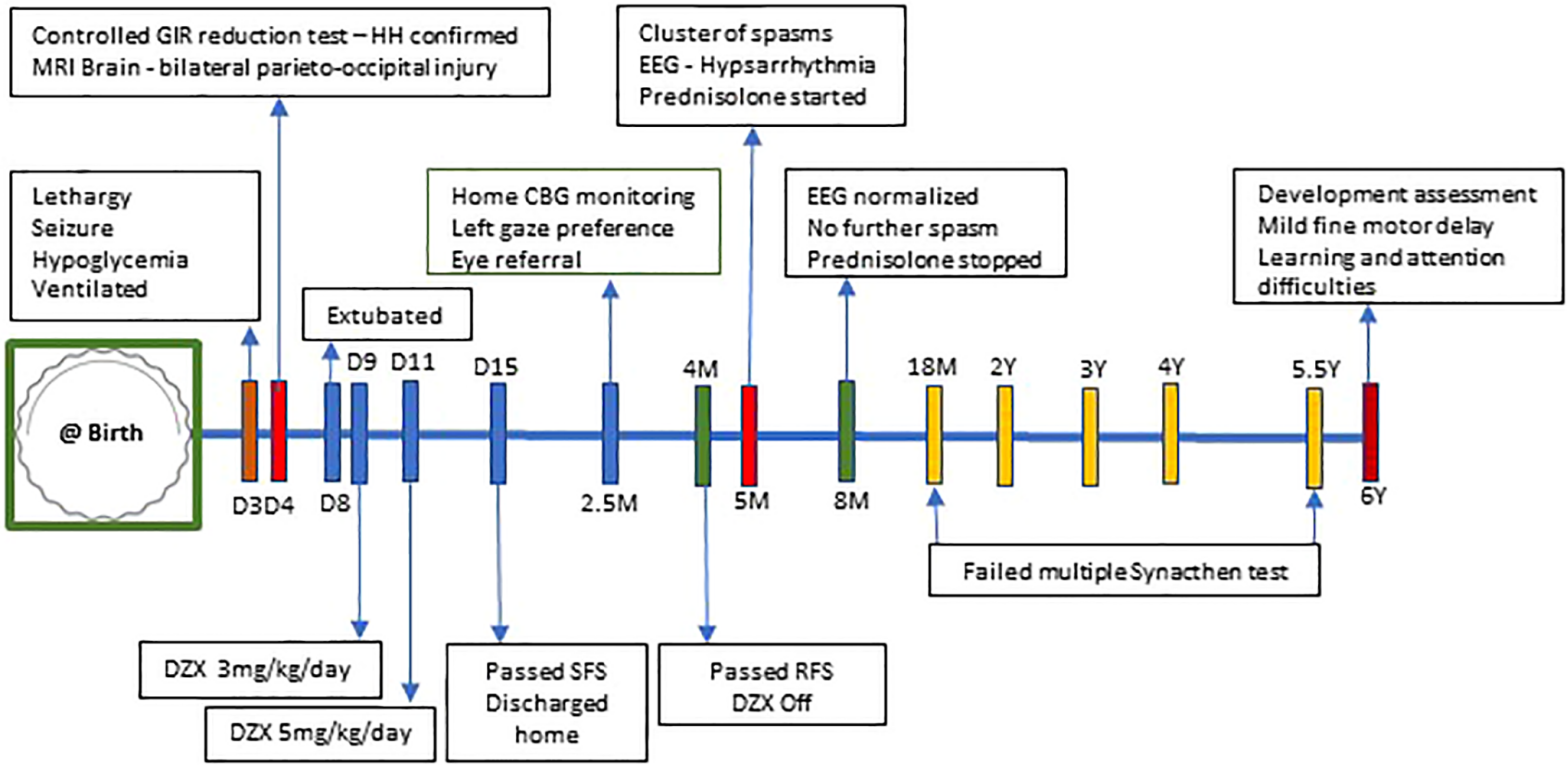
Timeline of events from day 3 of life to 6 years of age. CBG, capillary blood glucose; DZX, diazoxide; GIR, glucose infusion rate; HH, hyperinsulinemic hypoglycemia; RFS, resolution fast study; SFS, safety fast study; D, day of life; M, months; Y, years.

On follow-up, while on diazoxide at 2.5 months of age, he developed left gaze preference. He was referred to an ophthalmologist. Eye check was unremarkable and planned for review at regular intervals. But at the age of 5 months, he was hospitalized with new onset of clusters of spasms. The spasms were not associated with hypoglycemia. His biochemical and hematological investigations were within normal limits. The neurology team reviewed him, and an electroencephalogram (EEG) was done, which showed hypsarrhythmia (**Figure 3A**), in keeping with the diagnosis of IS. EEG recording of electrodecremental response during an episode of spasms was noted (**Figure 3B**). High-dose oral prednisolone (20 mg twice a day) was started for the treatment of IS. He remained asymptomatic after 48 h, and prednisolone was continued for 2 weeks and was weaned off over the next 4 weeks. A repeat EEG was normal. The patient appeared cushingoid clinically. In the background of HH and prolonged treatment with high-dose prednisolone, he was subjected to an ACTH stimulation test. Hypocortisolism, probably secondary to suppression of the hypothalamic–pituitary–adrenal axis, was noted. So oral hydrocortisone was advised. Serial ACTH stimulation tests were done at 18 months, 2 years, 3 years, 4 years, and 5.5 years of age and showed evidence of adrenal insufficiency. To date, he requires a physiological dose of hydrocortisone replacement and a stress dose regimen on sick days. He will have a repeat Synacthen test when he turns 7-year-old (**Figure 4**).

He is under close follow-ups with an ophthalmologist and a child developmental specialist. He has bilateral astigmatism and hypertropia, complicated with right eye amblyopia, for which he was prescribed glasses and advised left eye patching. Developmental screening assessment at 5 years 11 months of age using Strengths and Difficulties Questionnaire and Brigance Early Childhood Screen III (USA) have highlighted concerns in mainly 3 areas involving his learning, attention, and fine motor. He was started on school-based occupational therapy and psychological intervention, for which he had shown substantial improvement in his attention span and handwriting. He will be starting his Primary 1 in mainstream school next year.

## Discussion

We report a term SGA infant who sustained a parieto-occipital neuronal injury following profound HH on day 3 of life and who developed IS at age 5 months following the resolution of HH. While these features are neither new nor novel, two elements of clinical interest deserve further examination and discussion. The first pertains to the weight cutoff threshold of 2,270 g, which led to the missed opportunity to screen this infant and prevent hypoglycemia, while the second refers to gaze preference and its relationship with HH and IS. In addition, we performed a review of the pathophysiology and treatment of IS.

The cutoff value of 2,270 g was employed in our original hypoglycemia pathway as a result of Singapore’s developing past, which then provided a more realistic definition of a low birth weight (LBW) baby compared to the WHO’s value of 2,500 g. Based on a scale of 0 to 1, the Human Development Index (HDI) measures a country’s human development, where most developed countries score above 0.8. Although Singapore’s latest HDI is 0.938 (2019), it was 0.721 in 1990, reflecting Singapore’s developmental transition over 3 decades (8). Such a definition (2,270 g) of LBW for Singapore at the time appropriately avoided having a high incidence of LBW babies, which would create a heavy public health burden for developing countries (9) and may be relevant even today. Nonetheless, we recognized that 2,270 g was no longer meaningful and made adjustments to our clinical pathway in 2016 to reflect a more contemporaneous definition for LBW based on gestational age. This new pathway adopts a systems approach to prevent hypoglycemia in infants at risk (10).

HH is well known to cause NHBI, leading to epilepsy, developmental delay, and intellectual disability (3, 11). Neuronal deprivation of glucose causes impairment of the superficial cortex, dentate gyrus, hippocampus, caudate, and putamen mediated through several putative mechanisms (5). The onset, type, and cause of epilepsy among HH infants are variable and largely are due to occipital injury leading to IS (12). Although congenital structural abnormalities, including tuberous sclerosis, cortical malformations, congenital infections, and genetic syndromes, may cause IS, an acquired cause is more likely, such as hypoxic–ischemic injury or NHBI, while only a minority of IS is classified as idiopathic. In 2004, Camurdan et al. reported the first case of IS following HH (13). Subsequently, case reports and cohort studies linking NHBI and IS appeared in the literature (7, 14–17), as summarized in **Table 1**. Interestingly, none of these reports observed gaze preference as an antecedent to IS, as noted in our case.

**Table 1:** A summary of cohort studies of infants with neonatal hypoglycemic brain injury, infantile spasms, and neurodevelopmental outcomes.

Year of publication	Type of study	MRI findings	EEG	Outcome
Kumaran et al., 2010 (14)	Case series – 5 cases	NHBI—right parieto-occipital cyst in 1, rest normal	Hypsarrhythmia in 3, bilateral epileptic activity in 2	Speech and language delay in 2, gross motor and/or visual delay in 3
Yang et al., 2016 (15)	Cohort study – 18 cases	NHBI—bilateral or unilateral parietal and occipital changes in 55%	Hypsarrhythmia in all	No treatment or long-term outcome reported
Jia et al., 2017 (16)	Cohort study – 21 cases	NHBI—occipital cortex softening or glial scar in 76%	Hypsarrhythmia in all	Seizure control in 4.76%. Poor neurodevelopmental outcomes in all
Suranna et al., 2020 (7)	Retrospective cohort study – 113 cases	NHBI in 36% of IS cases. 90% with bilateral occipital injury	EEG not done	GDD in 95%
Kapoor et al., 2020 (17)	Retrospective cohort study – 170 cases	NHBI—gliosis in occipital or parietal lobe in 96.5%	Classical hypsarrhythmia in 49.4%, variants in 27.1%	GDD in 91.2%, cerebral palsy in 48.7%

NHBI, neonatal hypoglycemic brain injury; EEG, electroencephalogram; GDD, global developmental delay.

Gaze preference has been reported as the only symptom in occipital lobe epilepsy, without clinical seizures or altered consciousness (18). Excitation of the temporal–parietal–occipital cortex leads to epileptic gaze deviations. Shibata et al. reported a case of occipital lobe epilepsy presenting with left paroxysmal gaze deviations as the sole manifestation, which correlated with EEG, MRI, and single-photon emission CT imaging evidence of right occipital lobe hypoperfusion. The authors recommended doing video EEG studies on patients with frequent horizontal tonic eye deviations (18). In our patient, left gaze preference was first observed at 2.5 months before the onset of clinical spasms at 5 months of age. The unremarkable ophthalmology assessments from gaze preference to the development of spasms suggested possible subclinical or intermittent occipital epilepsy. These indicate the need to suspect subclinical IS when managing HH infants with preexisting occipital injury.

Researchers have tried to link SGA with IS through prenatal stress. There is growing evidence of the negative effect of prenatal stress on fetal growth in SGA infants (19) and HH (20). Animal experiments demonstrated that prenatal stress could increase fetal glucocorticoid levels, suppressing the hypothalamic–pituitary–adrenal axis and lowering cortisol levels after birth. This may explain the treatment response of IS to corticosteroids (21). Zou’s hypothesis, reported by Shi et al., proposed a “prenatal stress exposure hypothesis for IS” using animal models (22). Whether HH and IS manifestations are related to human fetal stress exposure is a subject that needs to be studied.

Hypoglycemia can cause profound cell apoptosis resulting in pontosubicular neuronal necrosis through activation of the caspase pathway (23). Several theories of neuronal cell deaths following hypoglycemia include activation of neuronal glutamate receptors contributing to excitotoxicity, oxidative stress with increased reactive oxygen species, mitochondrial dysfunction due to excessive neuronal zinc release, and extensive Poly-ADP-Ribose Polymerase-1 activation causing mitochondrial damage (24). Alkalay et al. reported occipital lobe involvement in MRI of 82% of 23 infants with profound hypoglycemia and half of them with visual impairment (25). Several other hypotheses link NHBI to IS. The brain maturation process evolves from occipital to frontal, with myelination of the occipital lobe appearing by 150–180 days postnatally, earlier than the rest of the brain (26). Observational studies by Hamano et al. and Endoh et al. have found a strong correlation between occipital region involvement and IS (27, 28). The visual cortex is the optical processing center located in the occipital lobe. The visual system undergoes considerable maturation after birth, with geniculostriate fibers migrating through the cortex (29). It has been hypothesized that the migrating optical fibers are unusually glucose-sensitive. Neuroglycopenia disrupts the temporal sequence of visual development, deprives cortical neurons of geniculostriate fibers, and impairs the maturation of the neurotrophic factors (30). This can lead to the classical findings of complete cortical absence or thinning in the parieto-occipital area. In 25 cases, Endoh et al. observed EEG findings of epileptic discharge in the occipital lobe before the onset of hypsarrhythmia and IS between 4 and 6 months of age (28). These findings support the development of IS in those who suffered a hypoglycemic occipital brain injury in the neonatal period. The predisposition of the occipital lobe to NHBI and the consistent finding of occipital lobe involvement in IS may help explain the link between NHBI, gaze preference, and IS, as portrayed in the index case.

Even though hormones (prednisolone or adrenocorticotrophic hormones) and vigabatrin are used to treat IS, current evidence supports using the former in the initial control of seizures (7, 31). Both vigabatrin and steroids have serious adverse effects. However, vigabatrin can cause significant irreversible retinal toxicity in up to 34% of cases, which is a cause of concern in infants with preexisting occipital neuronal injuries (32, 33). The UKIS study and Cochrane systematic review on the treatment of IS had determined hormonal therapy as first-line therapy as spasms resolved in more infants than those treated with vigabatrin and recommended the latter for treating IS in tuberous sclerosis (31, 34). Early diagnosis and treatment of IS have been consistently reported to have superior outcomes in response to treatment and developmental outcomes (7). Our infant responded well to high-dose oral prednisolone following a clinical diagnosis of IS in infancy. Although he has some learning difficulties, his ability to qualify for mainstream school gives him optimism.

### Strengths and Limitations

The strength of our case study lies in the detailed longitudinal phenotypic characterization of an SGA infant with HH, parieto-occipital injury, gaze deviation, IS, and neurocognitive delay. Parental understanding of the neurocognitive consequences after HH diagnosis and occipital injury led to the timely diagnosis of IS and initiation of hormonal therapy. Early diagnosis and treatment gave a good outcome at 6 years, enabling his admittance to mainstream school. We acknowledge a higher index of suspicion could have enabled an earlier etiological diagnosis for the gaze preference, even though formal pediatric ophthalmology referral was prompt. With the current knowledge of gaze preference predating occipital lobe epilepsy, a video EEG at the point of diagnosis of gaze abnormality at 2.5 months of age may have identified an occipital epileptic focus that could have been treated to prevent the development of IS.

## Conclusion

It is reasonable to anticipate neuronal injury in infants with symptomatic hypoglycemia, and MRI is paramount in documenting the brain insult. Once the parieto-occipital neuronal injury is noted in MRI, parents should be counseled to watch for seizures in the future. Long-term neurodevelopmental follow-up is recommended, including gaze assessment. A video EEG in infants with known neuronal injury presenting with gaze preference may be worthwhile. With the clinical presentation of IS, the aim should be to confirm the diagnosis using EEG and initiate early treatment. As seen in our case, hormonal therapy is preferred in acquired structural brain defects. If resistant to hormonal therapy in 2–4 weeks, vigabatrin is indicated. Overall, the outcome of our reported child was favorable due to timely diagnosis and early treatment of IS.

## Family’s Perspective

As parents, we were in shock when fits and low sugars happened so early in our son’s life. We were concerned about the MRI changes in his brain. We were fearful when he developed problems with his vision and then more fits even though sugar levels were normal. We are thankful to the doctors for the quick diagnoses, treatment, and regular follow-up and even more grateful that our son has improved to qualify for mainstream school.

## Data Availability Statement

The original contributions presented in the study are included in the article/supplementary material. Further inquiries can be directed to the corresponding author.

## Ethics Statement

Written informed consent was obtained from the minor(s)’ legal guardian for the publication of any potentially identifiable data included in this case report.

## Author Contributions

SC and FY treated the patient and wrote the final manuscript with appropriate references. Both authors contributed significantly to the scholarly content. KWT wrote the preliminary draft and prepared the figures. KJ contributed to the diagnosis and treatment of infantile spasms and reviewed the manuscript. All authors approved the final manuscript as submitted and agreed to be accountable for all aspects of the work.

## Conflict of Interest

The authors declare that the research was conducted in the absence of any commercial or financial relationships that could be construed as a potential conflict of interest.

## Publisher’s Note

All claims expressed in this article are solely those of the authors and do not necessarily represent those of their affiliated organizations, or those of the publisher, the editors and the reviewers. Any product that may be evaluated in this article, or claim that may be made by its manufacturer, is not guaranteed or endorsed by the publisher.
